# Cooperativity Model for Improving the Walking-Assistance Efficiency of the Exoskeleton

**DOI:** 10.3390/mi13071154

**Published:** 2022-07-21

**Authors:** Jianfeng Ma, Decheng Sun, Yongqing Ding, Daihe Luo, Xiao Chen

**Affiliations:** 1 Department of Materials and Manufacturing, Beijing University of Technology, Beijing 100124, China; majianfeng@bjut.edu.cn (J.M.); decheng1002@yeah.net (D.S.); dyq1656374401@163.com (Y.D.); luodaihedave@163.com (D.L.); 2 Systems Engineering Institute, Academy of Military Science, Beijing 100010, China

**Keywords:** cooperativity model, offset theory, passive walking-assistance exoskeleton, dispersion degree algorithm, energy conversion

## Abstract

(1) Background: To enhance the walking-assistance efficiencies of exoskeletons, this paper proposed the biomechanical-based cooperativity model based on a passive exoskeleton prototype to fill the technical gap in exoskeleton design regarding the torque transmission law between humans and exoskeletons. (2) Methods: The cooperativity model was used to solve the key system parameters based on the minimum average dispersion degree, in which the average dispersion degree algorithm based on the joint angle was designed and applied. (3) Results: The influence of the cooperativity model on the exoskeleton was indicated by comparing the walking-assistance efficiencies of the exoskeletons with the same structure but with different elastic parameters of the energy storage components, in which the exoskeleton based on the cooperativity design exhibited the highest walking-assistance performance. The walking-assistance efficiency of the exoskeleton with the optimal parameter combinations was also tested by comparing the respiratory metabolisms with and without the exoskeleton, in which the exoskeleton provided the average walking-assistance efficiency of 14.45% for more than 80% of the subjects. (4) Conclusions: The effects of the cooperativity model on exoskeletons were proven, but the accuracy and efficiency of the model still have room for improvement, especially the accuracy of the offset principle.

## 1. Introduction

The effective utilization of human energy is a hot research topic in recent years, such as storing the backpack-impact energy [1,2], the negative work of the human joint [3,4], and the heel-strike energy [5,6]. The exoskeleton is one of the research directions and its characteristic is to decrease the energy cost of the wearer by sharing their load and providing them with walking assistance [7]. It can be divided into two categories, powered exoskeletons and passive exoskeletons, according to whether external energy is needed.

Tucker et al. [8] proposed a general control framework that includes a three-layer hierarchical controller, in which the powered control is based on different control algorithm strategies and various sensors to capture specific trigger signals by the advanced controller, and then drive hydraulic, electrical, and pneumatic actuators to complete the walking-assistance function for the human body [9]. The ways to extract the motion intention of the wearer include sensitivity amplification control [10], model-based control [11], adaptive oscillator-based control [12], predefined gait trajectory control [13], fuzzy controller [14], mixed-assist strategy [15], and predefined actions based on gait mode [16]. At present, the development restriction of the powered exoskeleton is the energy storage density of the battery, which greatly weakens its application advantages in harsh environments.

Compared to the powered exoskeleton, the passive exoskeleton does not have some unstable factors, such as endurance and motion recognition, that the powered exoskeleton must deal with; the passive exoskeleton does not provide additional energy to the human body, but realizes the rational distribution of the human energy [17]. It provides two types of walking assistance for the human body.

The first way is that it is used to share the body weight of the wearer to reduce their gravity sense. The seat-shaped exoskeletons are used to support the body weight [18,19]. Exoskeletons with gravity compensation functions have proven to have a significant effect on patients with muscle weakness caused by stroke hemiplegia [20,21,22]. This walking-assistance method that does not consider the work of human muscles essentially defaults to the rule that gravity does the negative work in the process of lifting the system centroid [20,21,22]. The second way is that the exoskeleton partially replaces the muscles to drive the movement of the limbs. Ankle exoskeletons aim to provide the assisting torque to the flexion movement of the ankle joint. The joint angle [3] and plantar pressure [23] can be used as the basis for the design of the exoskeleton clutch. Van Dijk et al. [4,24] designed an exoskeleton with an artificial tendon, which is mainly used to transfer the energy between the multiple joints to decrease the energy cost of the wearers. The coupling between the different joints determines the energy interaction between the different joints.

Passive exoskeletons have advantages such as convenience, stability, and low cost, but their walking-assistance efficiencies are generally lower than powered exoskeletons [25,26]. Considering the working mechanism of the passive exoskeleton with the walking-assistance function [3,4,20,21,23,24], an important way to improve its walking-assistance efficiency is to improve the energy reuse efficiency [3,27]. In the existing exoskeleton [20,21,22,23,24], the macroscopic design of the exoskeleton is unilaterally emphasized, such as the walking-assistance period and working principle of the exoskeleton, while the microscopic design of the exoskeleton is ignored, especially the walking-assistance degree of the exoskeleton, which easily causes the exoskeleton to fail to adapt to the change of the joint torque, and even causes the exoskeleton to provide excessive torque to the human body, forcing the walking-assistance effect of the exoskeleton to become the resistance to the human body, thereby reducing the efficiency of the energy redistribution of the exoskeleton to the human body.

It is easy to understand that the reasonable walking-assistance degree can produce the best walking-assistance efficiency [28]. The powered exoskeleton outputs the matching torque to the human by monitoring the human physiological data [28,29], but this poses a challenge to the passive exoskeleton, which does not have real-time-control capabilities. Literature [25] analyzed the existing passive exoskeleton and concluded that the spring with the optimal stiffness is of great significance for the metabolic benefits of the exoskeleton. Literature [3] also found, in the test of a passive ankle exoskeleton, that there is an optimal spring stiffness that could produce the optimal metabolic benefits of the exoskeleton. The reasonable selection of the elastic coefficient of the exoskeleton system to enhance the metabolic benefits of the passive exoskeleton is the consensus of the existing research, but the selection mechanism of the optimal spring stiffness is not clear [3,23,25], especially the selection factors that affect the optimal stiffness, such as structural characteristics, walking-assistance principle, etc. To address this issue, in this paper, the cooperativity model was established to emphasize the research of the torque transmission law between humans and exoskeletons in torque cooperativity and the motion response of the exoskeletons in motion cooperativity, aiming to attach the working mechanism of the exoskeletons to the biomechanical law to more effectively realize the distribution of human energy.

The article structure of this paper presents the characteristics of continuity. In Section 2.1, the basic principle of the exoskeleton design—the cooperativity model—is summarized. In Section 2.2, the first step of the torque cooperativity—the offset principle—is described. In Section 2.3, the exoskeleton structure which reflects the motion cooperativity and the offset principle is described. In Section 3 and Section 4, the second step of the torque cooperativity—the parameter solution of the exoskeleton—is described, in which the solution process of the torque coordination is introduced, the average dispersion degree algorithm is designed and applied, and the concepts of three walking-assistance efficiencies (target walking-assistance efficiency, limit walking-assistance efficiency, and actual walking-assistance efficiency) and their relationship with each other are described. Finally, to validate the availability of the cooperativity model, the dynamic simulation of the exoskeleton and the respiratory metabolism test were respectively carried out in Section 5 and Section 6, in which this paper established and applied the formula for calculating the limit walking-assistance efficiency.

## 2. Exoskeleton Structure Design Based on the Cooperativity Model

### 2.1. Framework of the Cooperativity Model

In this paper, the exoskeleton design based on the cooperativity model was emphasized, and its essence is to realize the cooperativity between the exoskeleton and the human. In addition, the research for passive exoskeletons was highlighted in this article because when following the human movement it realizes the energy interaction with the human through the passive energy storage element with the monotonic and linear tension changes. During the tension-changing process, the working torque of the exoskeleton (WTE) to the human body has a functional relationship with the joint motion, and in the exoskeleton, there is no additional motion input and the complex torque change is easily realized by the powered exoskeleton. Therefore, the cooperativity model of the passive exoskeleton is mainly to constrain the motion response inside the exoskeleton and the original variation of the tension of the energy storage element.

**Figure 1:**
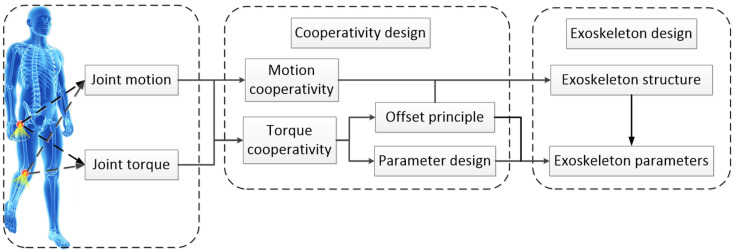
Process of the cooperativity model.

It is demonstrated in Figure 1 that the cooperativity model consists of the motion cooperativity and the torque cooperativity, in which the torque cooperativity includes the offset principle and the parameter design. The main content of the motion cooperativity is to achieve the motion consistency between the human and the exoskeleton, in which the exoskeleton has a reasonable motion response to ensure its predetermined function under the determined motion input of the human to the exoskeleton, including the design of the working mechanism of the energy storage element, the clutch mechanism in the exoskeleton, and the motion freedom of the exoskeleton.

The torque cooperativity is the research for the WTE. Firstly, to adapt the monotonic variation of the passive energy storage components to the complex variation of the joint torque, this paper proposes the offset principle to plan the overall trend of the WTE from the overall perspective, finding the appropriate gait interval to create the conditions for the intervention of the exoskeleton. The main task is to plan the action interval of the exoskeleton through a comprehensive understanding of the joint torque and the joint motion. At the same time, the offset principle qualitatively characterizes the working principle of the exoskeleton, realizing the rational distribution of energy in a single joint—from the equivalent dimension of force to partially or completely replace the joint torque. Secondly, to clarify the tension variation of the passive energy-storage components to further ensure that the WTE is close to the joint torque, this paper calculates the parameters of the energy storage components and the dimensions of the key structures of the exoskeleton to achieve the best walking-assisting effect on the human body. In particular, to select the optimal solution from many parameter combinations, an optimization algorithm based on biomechanics is proposed.

Therefore, as shown in Figure 1, the motion cooperativity defines the basic structure of the exoskeleton, and the offset principle defines the walking-assistance mode of the exoskeleton and affects the exoskeleton structure in detail. The parameter design is to make the final choice of the optimal solution of the elastic parameters based on the exoskeleton structure and the exoskeleton-walking-assistance mode to ensure the torque matching between the human and the exoskeleton, which reflects the influence of the exoskeleton structure factors, the exoskeleton-walking-assistance mode, the initial force of the spring, and the spring stiffness on the optimal spring stiffness.

### 2.2. Content of the Offset Principle

The function of the offset principle is to make the macro plan for the overall trend of the WTE under the determined motion input of the human body to the exoskeleton so as to create the framework for the parameter design in the next step. The apparent function of the exoskeleton is to achieve the rational distribution of human energy, but its essence is to replace the joint torque by exerting the reaction force on the human body. To facilitate the subsequent description, the joint motion direction was set as the macro-reference standard for the joint torque, in which the torque exerted along the direction of joint motion was defined as the joint positive work.

**Figure 2:**
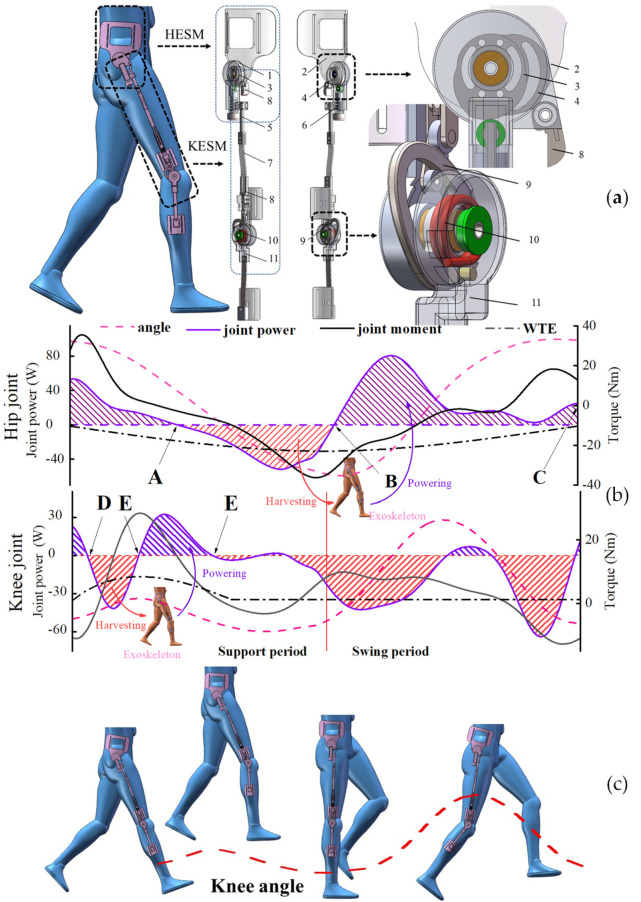
(**a**) Structure of the exoskeleton. 1—Hip shell cover, 2—Junction panel, 3—Main cam, 4—Minor cam, 5—Pushrod 1, 6—Pressure spring, 7—Pushrod 2, 8—Pushrod 3, 9—Cam, 10—Torsional spring, 11—Shell cover. (**b**) Movement characteristics of the lower limb joints. (**c**) Movement characteristics of the exoskeleton.

The specific exoskeleton structure establishes the function relationship between the reciprocating joint motion and the linear change of the tension of the passive energy-storage element, in which the same state of the joint in the reciprocating motion corresponds to the same state of the energy-storage element. Although the energy-storage element presents different energy-storage or energy-release states at this time, the reaction force of the exoskeleton on the human body is consistent. Therefore, the essential effect of the offset principle is to set a reasonable monotonic interval for the passive energy-storage element based on the joint torque and the joint motion, which means, finding the continuous intervals with the opposite joint motions and the opposite joint torques. The movement characteristics of the hip and knee joints are shown in Figure 2b.

For the hip joint, there are two continuous intervals satisfying the needs near node B—the negative torque should be provided for the hip joint before node B, and the positive torque should be provided after node B. From the perspective of energy, the joint negative work $\int_{t_{A}}^{t_{B}}P_{joint-power}(t)dt$ is recovered and compensated for the joint positive work $\int_{t_{B}}^{t_{c}}P_{joint-power}(t)dt$.

Due to the time when node A appears also being different between humans, the period before node A was also used to store energy in this paper. Although this will inevitably add a burden to the human body, the energy stored beyond the standard state will eventually be fed back to the human body. For the hip joint, the comparative relationship between the WTE and the joint torque is shown in Figure 2b. How to reduce the gap between the two torques requires the reasonable calculation for the relevant parameters in the hip energy storage mechanism (the HESM in Figure 2a), which is clarified in Section 3.

The knee joint exhibits variable stiffness characteristics throughout the gait cycle [30,31], so discrete assistance measures should be implemented for the knee joint. During the swing period, the knee energy storage mechanism (the KESM in Figure 2a) should not significantly interact with the knee joint and during the support period, the negative torque should be provided for the knee joint between nodes C and D, and the positive torque should be provided between nodes D and E. From the energy perspective, the joint negative work $\int_{t_{D}}^{t_{E}}P_{joint-power}(t)dt$ is recovered and compensated for the joint positive work $\int_{t_{E}}^{t_{F}}P_{joint-power}(t)dt$ (Figure 2b). For the KESM, reducing the gap between the WTE and the joint torque is elucidated in Section 4.

### 2.3. Exoskeleton Prototype for Solving the Cooperativity Model

To further describe the solving process of the cooperativity model, a passive exoskeleton was applied in this paper (Figure 2a). The HESM and the KESM are the modular names of the exoskeleton serving the human hip and knee joints, respectively. Each mechanism has a knob to adjust the initial torque or initial force of the elastic element to depersonalize their walking-assistance effect.

#### 2.3.1. Structure of the HESM

To realize the requirement of the offset principle for the HESM, the mechanism was designed and is shown in Figure 2a. The superposition of the main cam and the minor cam is fixed to the junction panel. The build-in reset torsion spring of the pawl avoids the influence of gravity on the pawl to ensure that the pawl produces timely motion interference to the roller follower under the motion input of the minor cam. The energy storage and release of the HESM are achieved by defining the compression law of the main cam to the pressure spring. When the hip joint swings backward, the main cam continuously completes the compresses for the pressure spring, in which the negative work was recycled by the HESM. When the hip joint swings forward, the compression of the pressure spring is continuously reduced, in which the KESM feedbacks the recovered energy to the joint positive work.

#### 2.3.2. Structure of the KESM

To realize the discretized assistance requirement of the offset principle for the knee joint, the KESM with one DOF was designed and is shown in Figure 2a. The KESM utilizes the design method of the clutch mechanism in the motion cooperativity, using the motion response of the exoskeleton hip joint to control the motion response of the exoskeleton knee joint. As shown in Figure 2c, when the main cam compresses the pressure spring, through the rolling friction force the minor cam drives the pawl rotation to constrain the upward-lifting of the roller follower, and further achieve the energy-storage control for the KESM. The torsional spring is a component that transmits torque between the shell cover and the cam (Figure 2a). Under the position constraint of the pawl, the roller follower further restricts the clockwise rotation of the cam, so the deflection of the torsional spring is equal to the motion angle of the knee joint, during which the HESM completes the energy interaction with the human body.

## 3. Torque Cooperativity Solution for the HESM

**Figure 3:**
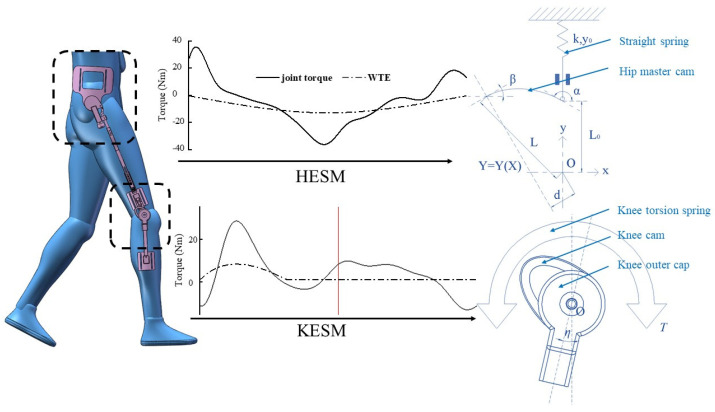
Mechanism schematic diagram of the HESM.

The working principle of the HESM is to achieve the human–exoskeleton energy transmission in the dynamic change of the compression degree of the main cam to the pressure spring. The mechanism achieves the distribution of human energy by changing the joint torque, in which the walking-assistance function of the exoskeleton is realized. So, the scientific judgment for the mechanism requires the comparative analysis between the joint torque and the WTE, and its essential significance is to make the reasonable design for the elastic parameters of the energy storage components and the cam profile of the main cam. Under the definition of the offset principle, the comparison concerning the WTE of the HESM and the joint torque is shown in Figure 2b and Figure 3. The main content of this Section is to reduce the gap between the two under certain scaling conditions.

The simplified physical model of the cam roller mechanism is shown in Figure 3, in which k, $y_{0}$, α, β, d, $L_{0}$, and L represent respectively the spring stiffness, the initial spring compression, the slope of the cam curve at the initial point, the slope of the cam curve at the end point, the force arm of the cam, the radius of the cam base circle, and the distance between the endpoint of the cam curve and the rotation center. To facilitate calculation and analysis, this paper set the curve equation as a cubic function based on the lowest four unknown quantities. After considering the influence factors for the moment arm d and the size of the mechanism, $L_{0}$, L, and β were selected as 20 mm, 30 mm, and 0 deg respectively. α, k, and $y_{0}$ lack of the established basis, which needs to be obtained by guaranteeing the maximum walking-assistance effect of the mechanism. The cam profile and its tangent equation are shown in Equation (1).

(1) $$\begin{array}{l} y=Ax^{3}+Bx^{2}+Cx+D\\ y^{\prime }=3Ax^{2}+2Bx+C \end{array}$$

We bring L=30, β=0, $L_{0}=20$, and α into Equation (1) and simplify the equations to obtain Equation (2).

(2) $$\{\begin{array}{c} y=Ax^{3}+Bx^{2}+Cx+D\\ A=-\frac{4}{30^{4}}(30^{2}-\frac{1}{2}30^{2}\tan(\alpha )-600\sqrt{2})\\ B=-\frac{4}{30^{4}}(\frac{\sqrt{2}}{2}30^{3}\tan(\alpha )-\frac{3\sqrt{2}}{4}30^{3}+30^{3})\\ C=\tan(\alpha )\\ D=20 \end{array}$$

We define Equation (3), which represents the distance from the point (x,y) to the origin O.

(3) $$\begin{array}{l} l=l(x,y(x))=\sqrt{x^{2}+y^{2}}\\ l^{\prime }=(\sqrt{x^{2}+y^{2}}{)}^{\prime }=-\frac{2x+2y(y^{\prime })}{2\sqrt{x^{2}+y^{2}}}=-\frac{x+(Ax^{3}+Bx^{2}+Cx+D)(3Ax^{2}+2Bx+C)}{\sqrt{x^{2}+{\left(Ax^{3}+Bx^{2}+Cx+D\right)}^{2}}} \end{array}$$

**Figure 4:**
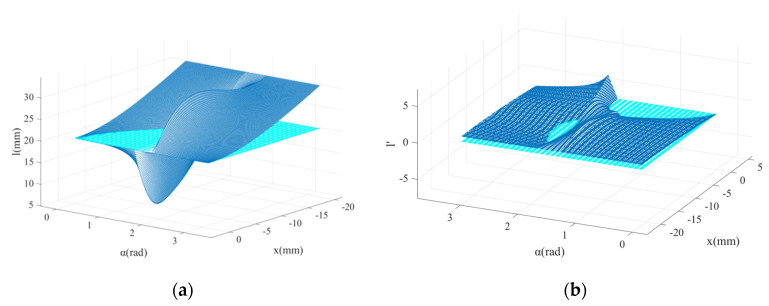
(**a**) Profile of the main cam with different α; (**b**) derivative diagram of the profile of the main cam with different α.

To avoid tan(α) tending to infinity, after taking $x\subset (-\sqrt{2}L/2,0)$, α⊂(0,0.45Π)∪(0.6Π,Π) the three-dimensional graph of l=l(α,x) and $l^{\prime }=l^{\prime }(\alpha ,x)$ are obtained and shown in Figure 4, in which the cyan planes (l=0 and $l^{\prime }=0$) are the isosurface and are used as the reference planes.

Obviously, in the case of the monotonous change of x, the same change of l is not allowed; the compression of the cam on the spring must maintain monotonicity to ensure the monotonic performance of the spring. From the perspective of the curve equation, α should be valued in the interval (1.92, 3.14). It is worth noting that when α tends to be 1.92, in the x-direction, the change rate of $l=l^{\prime }(\alpha ,x)$ is larger in the middle section and smaller at both ends.

As shown in Figure 3, the normal equation of the cam profile is defined as Equation (4).

(4) $$Y=(-\frac{1}{y^{\prime }})\left(X-x\right)+y$$

Further, the force arm of the reaction force of the pressure spring to the main cam is shown in Equation (5).

(5) $$d=\frac{\left|y+(\frac{1}{y^{\prime }})x\right|}{\sqrt{(\frac{1}{y^{\prime }}{)}^{2}+1}}$$

Equation (6) can be obtained by substituting y and $y^{\prime }$ into Equation (5).

(6) $$d=\frac{\left|(Ax^{3}+Bx^{2}+Cx+D)+\frac{1}{\left(3Ax^{2}+2Bx+C\right)}x\right|}{\sqrt{{\left(\frac{1}{3Ax^{2}+2Bx+C}\right)}^{2}+1}}$$

**Figure 5:**
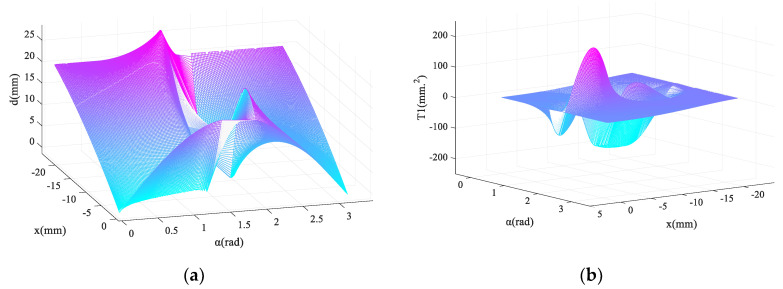
(**a**) Moment arm of the hip master cam; (**b**) three-dimensional diagram of $T_{1}$.

Similarly, after taking $x\subset (-\sqrt{2}L/2,0)$, α⊂(0,0.45Π)∪(0.6Π,Π), the three-dimensional graph of d(α,x) can be obtained (Figure 5a).

When α tends to be 3.14, the moment arm has a smooth transition and the result becomes more valuable. Further, the output torque equation of the mechanism is shown in Equation (7).

(7) $$\begin{array}{l} T=kT^{\prime }=k(y-y(0)+y_{0})d=k((Ax^{3}+Bx^{2}+Cx)d+y_{0}d)=T_{1}+T_{2}\\ =k(Ax^{3}+Bx^{2}+Cx+y_{0})\frac{\left|(Ax^{3}+Bx^{2}+Cx+D)+\frac{1}{\left(3Ax^{2}+2Bx+C\right)}x\right|}{\sqrt{{\left(\frac{1}{3Ax^{2}+2Bx+C}\right)}^{2}+1}} \end{array}$$

In the above formula, T is the superposition of $T_{1}$ and $T_{2}$, where $T_{2}$ represents the scaling of d(α,x) from the numerical perspective, which is the clarified change. Then it is advisable to judge the variation of $T_{1}=T_{1}\left(\alpha ,x\right)$, and its three-dimensional diagram with k=1 is shown in Figure 5b. The changes of $T_{1}$ and $T_{2}$ are similar, and the main function of $y_{0}$ is to control the occupancy of the basic stable quantity $T_{2}$ in T. Therefore, $y_{0}=5$ is taken to ensure the performance stability of the mechanism.

For a certain α that meets the design requirement range, the extreme value of T along the x-direction is delayed as the α increases. The value of α and k need to be selected according to the joint torque of the hip joint. However, the excessive pursuit for the similarity between the WTE of the HESM and the joint torque will lead to the interference of the exoskeleton for the original exercise habits of the human body. The effective walking-assistance method of the exoskeleton is to partially compensate for the joint torque. In this paper, the appropriate values of α and k were selected according to the dispersion degree between the reduced joint torque and WTE under each value of α and k. Human kinematics data [32]—joint torque, joint angle, and joint power—are presented together based on the gait process as the dependent variable. However, the gait process is based on the whole gait cycle as the research background, which has a certain macroscopic significance and is not conducive to direct the mathematical derivation. Therefore, this paper established the relationship between the WTE and the joint torque with the joint angle as the dependent variable.

**Figure 6:**
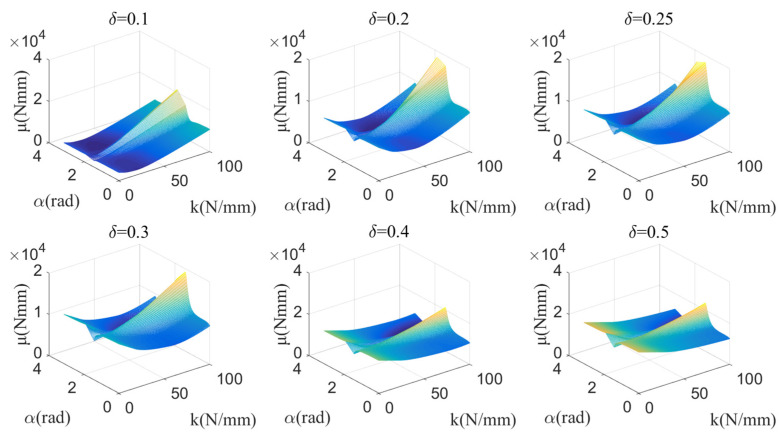
Dispersion value of the HESM under different parameters.

The average dispersion degree algorithm based on the relation between the joint motion and the joint torque is shown in Equation (8), where μ(α,k), δ, T(α,k,x), $T_{swing-back}$, and $T_{swing-forward}$ represent the average dispersion degree between the joint torque and the WTE, the scaling ratio of the joint torque, the WTE, the hip joint torque with the swings—backward joint, and the hip joint torque with the swings—and forward joint. Equations (8 (1)) and (8 (2)) were used to calculate the average dispersion of discrete curves and continuous curves respectively. Based on Equation (8 (2)), the calculation results for μ(α,k) under the different parameter values δ are shown in Figure 6.

**Figure 7:**
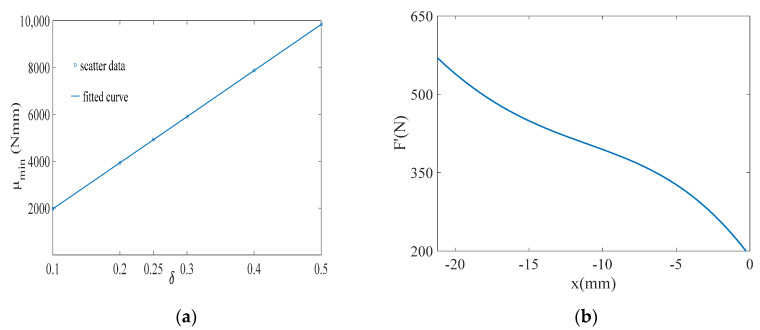
(**a**) $\mu_{\min}$ of each δ for the hip joint; (**b**) elastic force of the pressure spring.

The value of δ represents the target walking-assistance efficiency. Adjusting the value of δ within a certain range can help improve the walking-assistance efficiency of the exoskeleton, but whether there is a clear proportional relationship between the value of δ and the actual walking-assistance efficiency still needs further analysis. It is worth noting that in Section 5, the limit walking-assistance efficiency of the exoskeleton under the framework of the offset principle is calculated by defining Equations (13) and (15), which essentially predicts the limit interference between human and exoskeleton from the mathematic dimension and is beneficial to define the value range of δ. However, although the maximum limit of δ can be roughly calculated, the larger value should not be selected. As shown in Figure 7a, $\mu_{\min}$ of each δ are extracted from Figure 6, in which $\mu_{\min}$ represents the minimum dispersion the between the WTE of the HESM and the joint torque for each δ setting. Obviously, with the increase of δ, $\mu_{\min}$ also increases almost linearly, which means that the improvement of the walking-assistance efficiency brought by the increase of δ is gradually eroded by the increase of $\mu_{\min}$.

(8) $$\begin{array}{c} (1)\mu (\alpha ,k)=\frac{\int \left|T(\alpha ,k,\arctan(\frac{y(x)}{x}))-\delta T_{swing-back}(\arctan(\frac{y(x)}{x}))\right|+\left|T(\alpha ,k,\arctan(\frac{y(x)}{x}))-\delta T_{swing-forward}(\arctan(\frac{y(x)}{x}))\right|dx}{2\int 1dx}\\ (2)\mu (\alpha ,k)=\frac{\sum_{i=1}^{n_{1}}\left|T(\alpha ,k,\arctan(\frac{y(x(i))}{x(i)}))-\delta T_{swing-back}(\arctan(\frac{y(x(i))}{x(i)}))\right|+\sum_{i=1}^{n_{2}}\left|T(\alpha ,k,\arctan(\frac{y(x(i))}{x(i)}))-\delta T_{swing-forward}(\arctan(\frac{y(x(i))}{x(i)}))\right|}{n_{1}+n_{2}} \end{array}$$

According to Figure 6 and Figure 7a, δ=0.2 is selected, expecting to provide 20% assistance to the human body. When δ=0.2, $\mu_{\min}$ corresponds to k=38 N/mm and α=2.36 (α=2.36 satisfies the condition α⊂(1.92, 3.14)). The elastic force equation of the pressure spring is shown in Equation (9) and its change curve is shown in Figure 7b.

(9) $$F^{\prime }=(l+5)k=38(l+5)$$

## 4. Torque Cooperativity Solution for the KESM

The KESM applies the offset theory in the mutual mapping between the movement angle of the knee joint and the deformation of the torsional spring. In addition, as shown in Figure 2a, the initial torque of the torsional spring is a fixed value for a single mechanism—the initial torque is used to overcome the influence of gravity on the mechanism to achieve timely contact between the roller follower and the pawl under the drive of the torsional spring—and its value was set to 1150 Nmm. Then, based on human mechanics, the key to the optimal design of the KESM lies in the selection of the stiffness of the torsional spring. Under the definition of the offset principle, the comparison concerning the WTE of the KESM and the joint torque is shown in Figure 2b and Figure 3. The main content of this Section is to ensure the minimum dispersion between the two under certain scaling conditions.

**Figure 8:**
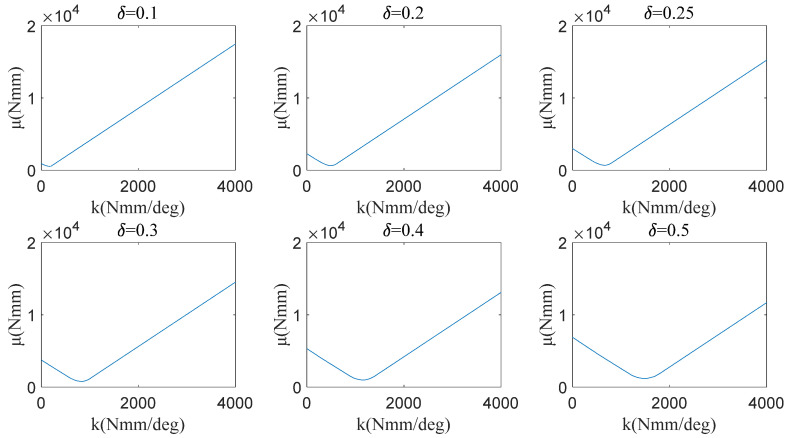
Dispersion value of the KESM under different parameters.

The joint torque at the knee joint references another paper [32]. The average dispersion degree algorithm based on the relation between the joint motion and the joint torque is shown in Equation (10), where $T_{0}$, k, η, μ(k), T(k,η), δ, $T_{swing-back}$, and $T_{swing-forward}$ represent the primary torque of the torsional spring (1150 Nmm), the stiffness of the torsional spring, the angle of the knee joint, the measures of the dispersion between the joint torque and the WTE, and the WTE, the scaling ratio of the joint torque, the joint torque when the knee joint is gradually bent, and the joint torque when the knee joint is gradually straightened. Equations (10 (1)) and (10 (2)) were used to calculate the discreteness of discrete curves and continuous curves respectively. Based on Equation (10 (2)), the calculation results for μ(k) under the different parameter values δ are shown in Figure 8.

(10) $$\{\begin{array}{c} (1)\mu (k)=\frac{\int \left|T_{0}+k\eta -\delta T_{swing-back}(\eta )\right|+\left|T_{0}+k\eta -\delta T_{swing-forward}(\eta )\right|d\eta }{2\int 1d\eta }\\ (2)\mu (k)=\frac{\sum_{i=1}^{n_{1}}\left|T_{0}+k\eta (i)-\delta T_{swing-back}(\eta (i))\right|+\sum_{i=1}^{n_{2}}\left|T_{0}+k\eta (i)-\delta T_{swing-forward}(\eta (i))\right|}{n_{1}+n_{2}} \end{array}$$

**Figure 9:**
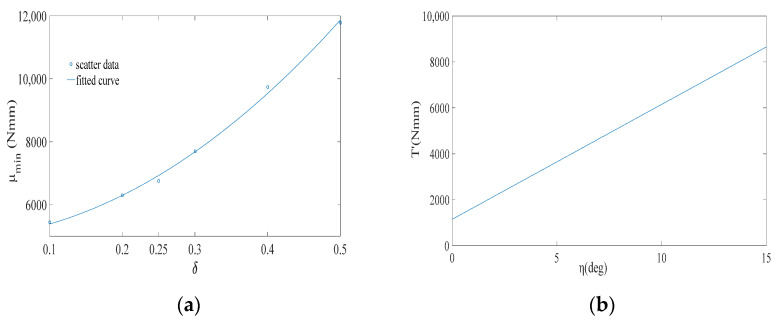
(**a**) $\mu_{\min}$ of each δ for the knee joint; (**b**) torque of the torsional spring.

As shown in Figure 9a, $\mu_{\min}$ of each δ are extracted from Figure 8, in which with the increase of δ, $\mu_{\min}$ also increases. Similar to the HESM, δ=0.2 is chosen for the KESM. When k takes the value of 500 Nmm/deg, the KESM has the smallest average dispersion. Taking into account the unidirectionality of the knee torque during the support period, 500 Nmm/deg is theoretically the best choice. The torque equation of the torsional spring is shown in Equation (11), and its curve is shown in Figure 9b.

(11) $$T^{\prime }=T_{0}+k\eta =1150+500\eta$$

## 5. Verification and Discussion on the Function of the Cooperativity Model

The influences of the elastic parameters of the energy storage components and the key structures of the exoskeleton for the exoskeleton were analyzed, and the optimal parameter combinations were selected for the HESM and the KESM respectively. Due to the lack of objective comparative conditions among all exiting exoskeletons, the effect of the cooperativity model on the exoskeleton was indicated by comparing the walking-assistance efficiencies of the exoskeletons with the same structure but with different elastic parameters of the energy storage components. The exoskeletons with the optimal parameter combinations were selected as the experimental group of the cooperativity model, and the exoskeletons with other parameter combinations were selected as the reference group. The exoskeletons with different design parameters were subjected to dynamic simulation to verify the validity of the cooperativity model. In addition, to ensure the objectivity and rationality of the simulation results, this paper refers to the simulation strategy adopted by another paper [17].

### 5.1. Effect of the Cooperativity Model on the HESM

**Figure 10:**
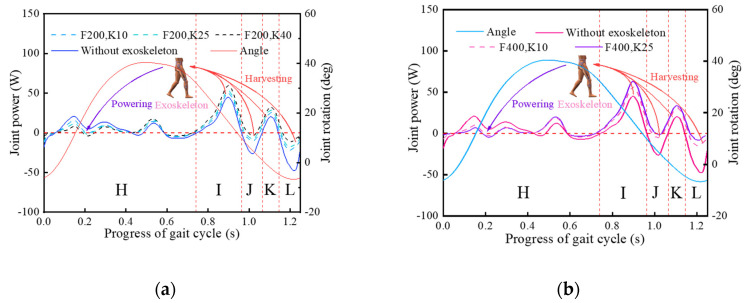
(**a**) Pressure spring with the initial compression force of 200 N; (**b**) Pressure spring with the initial compression force of 400 N.

To test the influence of different parameter combinations on the exoskeleton, this paper appropriately adjusted the analysis results in Section 3, selected 200 N and 400 N for the initial force, and 10 N/mm, 25 N/mm and 40 N/mm for the stiffness. The joint powers with the exoskeletons with different parameter combinations are shown in Figure 10, where F (N) represents the initial pressure of the pressure spring and K (N/mm) represents the stiffness of the pressure spring.

Based on Figure 10, in most regions of the whole gait cycle, it is obvious that the joint power with the exoskeleton is smaller than without the exoskeleton. Specifically, in the part area of the joint negative work, the decrease of the joint powers conforms to the walking-assistance characteristics of the exoskeleton. However, it must be affirmed that in the areas of the joint positive work in region A, the joint power increases instead, which conforms to the strategy adopted by the exoskeleton design for the transition uncertainty concerning the positive and negative work. It is worth noting that there is one anomalous issue that does not conform to the understanding of this paper. There is a positive work area near 1.1 s—the joint positive work in the collection area of the joint negative work violates the design principle of the mechanism [4,22,32]. This phenomenon, which is abnormal compared with the public data, needs to be further considered.

To have an objective evaluation of the working performance of the exoskeleton, this paper introduced a calculation formula to measure the overall distribution of the hip joint power, which is based on the consideration of the discrete data results. The formula based on the average is shown in Equation (12).

(12) $$\{\begin{array}{c} \bar{\eta }=\frac{\sum_{h=1}^{N_{{}_{H}}}\left|w_{h}\right|+\sum_{i=1}^{N_{{}_{I}}}\left|w_{i}\right|+\sum_{j=1}^{N_{{}_{J}}}\left|w_{j}\right|+\sum_{k=1}^{N_{K}}\left|w_{k}\right|+\sum_{l=1}^{N_{L}}\left|w_{l}\right|}{N_{{}_{H}}+N_{{}_{I}}+N_{{}_{J}}+N_{{}_{K}}+N_{L}}\\ N_{{}_{H}}+N_{{}_{I}}+N_{{}_{J}}+N_{{}_{K}}+N_{L}=N_{{}_{TN}} \end{array}$$

Further, the formula for the limit walking-assistance efficiency is defined in Equation (13).

(13) $$\{\begin{array}{c} \psi =100\%-\frac{\left|\frac{\sum_{h=1}^{N_{{}_{H}}}w_{h}}{N_{{}_{H}}}+\frac{\sum_{j=1}^{N_{{}_{J}}}w_{j}}{N_{{}_{J}}}+\frac{\sum_{l=1}^{N_{L}}w_{l}}{N_{{}_{L}}}\right|+\left|\frac{\sum_{i=1}^{N_{I}}w_{i}}{N_{{}_{I}}}\right|+\left|\frac{\sum_{k=1}^{N_{{}_{K}}}w_{k}}{N_{{}_{K}}}\right|}{\frac{\sum_{h=1}^{N_{H}}\left|w_{h}\right|}{N_{{}_{H}}}+\frac{\sum_{i=1}^{N_{{}_{I}}}\left|w_{i}\right|}{N_{{}_{I}}}+\frac{\sum_{j=1}^{N_{{}_{J}}}\left|w_{j}\right|}{N_{{}_{J}}}+\frac{\sum_{k=1}^{N_{{}_{K}}}\left|w_{k}\right|}{N_{{}_{K}}}+\frac{\sum_{l=1}^{N_{{}_{L}}}\left|w_{l}\right|}{N_{{}_{L}}}}\times 100\%\\ N_{{}_{H}}+N_{{}_{I}}+N_{{}_{J}}+N_{{}_{K}}+N_{L}=N_{{}_{TN}} \end{array}$$

In Equations (12) and (13), $w_{h}$,$w_{i}$,$w_{j}$,$w_{k}$, and $w_{l}$ represent respectively the discrete points of the interval *H*, *I*, *J*, *K*, and *L*, and $N_{{}_{TN}}$ is equal to the total number of all discrete point (Figure 10).

**Figure 11:**
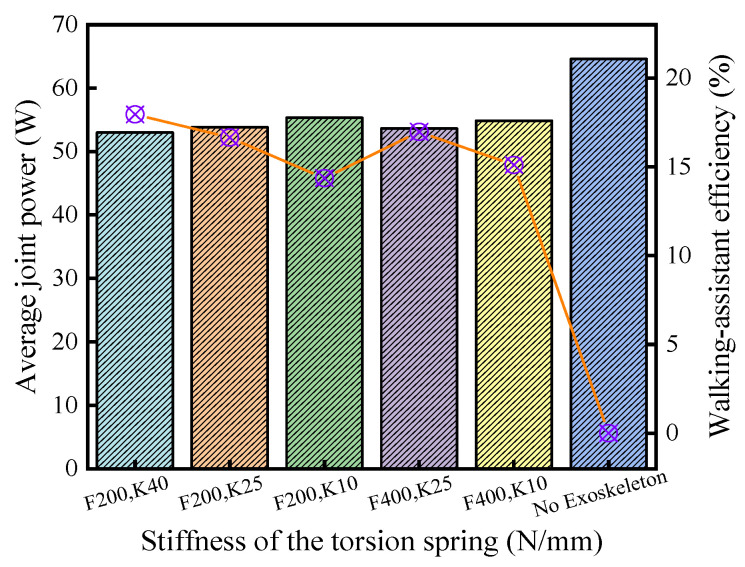
Walking-assistance efficiencies of the HESM.

Through further calculations, the walking-assistance efficiencies of the exoskeleton under the five parameter combinations are shown in Figure 11 and the results were 17.95%, 16.65%, 14.36%, 16.97%, and 15.10%, in which the HESM with the optimal parameter combinations exhibited the highest walking-assistance efficiency [3,25]. In addition, the walking-assistance efficiencies obtained by the dynamic simulation were slightly lower than the target walking-assistance efficiency, which is 20%, and generally higher than the previous lower-limb-assistant exoskeleton [3,4,23,24,30], but far lower than the extreme walking-assistance efficiency, which is 52.36%.

### 5.2. Effect of the Cooperativity Model on the KESM

**Figure 12:**
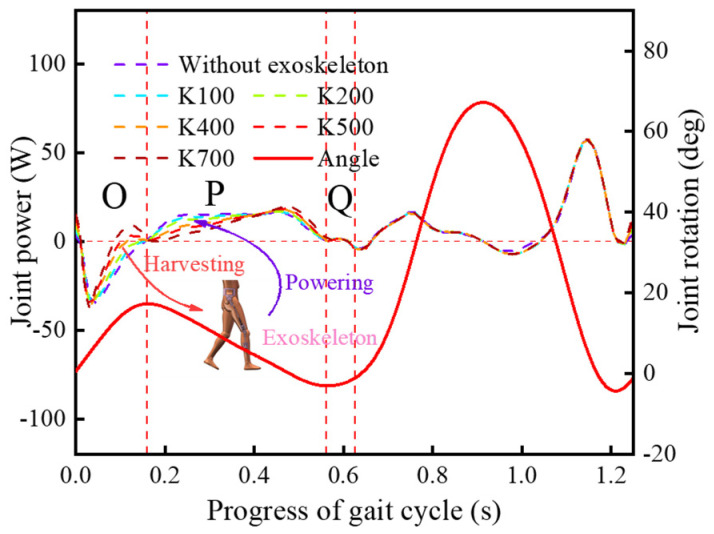
Joint power with the KESM under different stiffness values.

Compared with the hip joint, the discrete assistance measure was implemented for the knee joint [25,28]. The evaluation for the KESM is based on the support period, and 100 Nmm/deg, 200 Nmm/deg, 400 Nmm/deg, 500 Nmm/deg, and 700 Nmm/deg were selected for the spring stiffness. The joint powers of the exoskeletons with different spring stiffness are shown in Figure 12, where K (Nmm/deg) means the torsional rigidity of the torsional spring.

There were significant amplitude differences between the joint powers during the support period and the high similarity during the swing period [25,28]. This indicates that the KESM affects the body during the support period, and this interaction has a positive effect on the movement of the knee joint. To objectively judge the walking-assistance efficiency of the KESM, this paper introduced a calculation formula to measure the overall distribution of the knee joint power, which is based on the consideration of the discrete data results. The formula based on the average is shown in Equation (14), and the limit walking-assistance efficiency formula of the KESM is defined in Equation (15).


(14) $$\{\begin{array}{c} \bar{\eta }=\frac{\sum_{o=1}^{N_{O}}\left|w_{o}\right|+\sum_{p=1}^{N_{{}_{P}}}\left|w_{p}\right|+\sum_{q=1}^{N_{{}_{Q}}}\left|w_{q}\right|}{N_{O}+N_{P}+N_{{}_{Q}}}\\ N_{O}+N_{P}+N_{{}_{Q}}=N_{{}_{SP}} \end{array}$$

(15) $$\{\begin{array}{c} \psi =100\%-\frac{\frac{\sum_{o=1}^{N_{O}}\left|w_{o}\right|}{N_{{}_{F}}}+\frac{\sum_{p=1}^{N_{P}}\left|w_{p}\right|}{N_{{}_{G}}}+\frac{\sum_{q=1}^{N_{{}_{Q}}}\left|w_{q}\right|}{N_{{}_{H}}}}{\frac{\sum_{r=1}^{N_{{}_{SP}}}\left|w_{r}\right|}{N_{{}_{SP}}}}\times 100\%\\ N_{O}+N_{P}+N_{{}_{Q}}=N_{{}_{SP}} \end{array}$$

**Figure 13:**
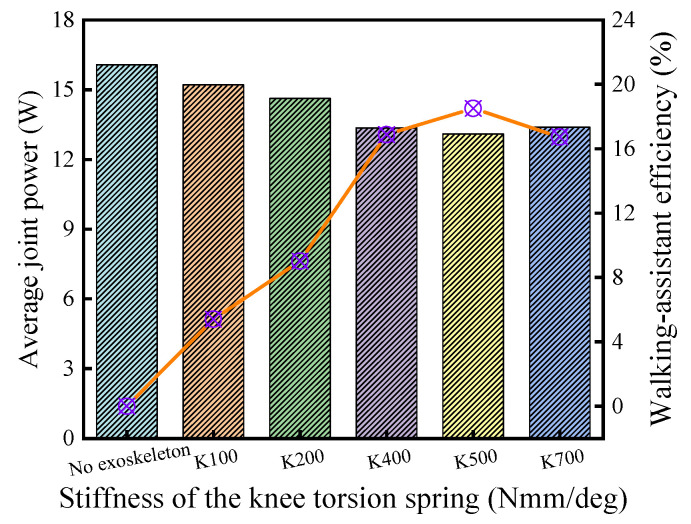
Walking-assistance efficiencies of the KESM.

In Equations (14) and (15), $w_{o}$, $w_{p}$, and $w_{q}$ represent respectively the discrete points of the interval *O*, *P*, and *Q*, and $N_{SP}$ represents the total number of the discrete points (Figure 12). The calculation results of each curve are shown in Figure 13, and the walking-assistance efficiencies were 5.41%, 9.02%, 16.88%, 18.51%, and 16.72%, respectively, in which the KESM with the optimal parameter combinations exhibited the highest walking-assistance efficiency [3,25]. The walking-assistance efficiencies of simulation are generally higher than the previous lower-limb-assistant exoskeleton [3,4,23,24,30,31] but far less than the theoretical limit of walking-assistance, which is 66.72%.

## 6. Respiratory Metabolism Test of the Exoskeleton Based on the Cooperativity Model

To test the walking-assistance efficiency of the exoskeleton with the optimal parameter combinations, the respiratory metabolisms with and without the exoskeleton were compared to calculate the efficiency, in which the gas metabolism instrument and the heart rate band were employed to measure the respiratory metabolism [30]. COSMED Gas Metabolism Meter calculates the energy consumption of the human body through the heart rate (HR), carbon dioxide emission ($VCO_{2}$), and oxygen consumption ($VO_{2}$). To ensure the unique variable, the subjects were required to complete a 30 min test on the treadmill with a constant speed of 4 Km/h after wearing the test equipment. At the same time, the subjects were required to participate in interaction with the researchers during the test, which could not only obtain the subjective feelings of the subjects in real-time but also attract their attention to stabilize their brain metabolism. The metabolism over the 2 min after the steady exercise was used for the data analysis due to the higher accuracy and representativeness of the metabolic test under steady exercise [24,33].

**Figure 14:**
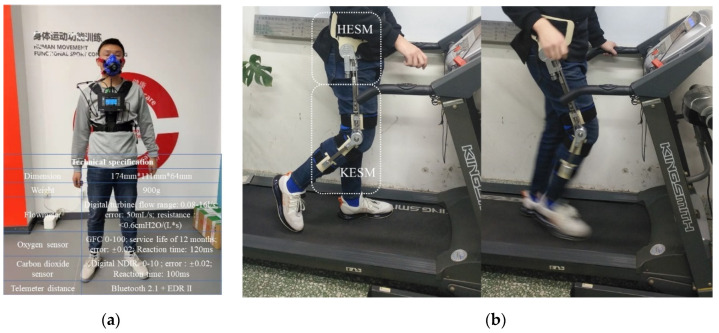
(**a**) COSMED Gas Metabolism Meter; (**b**) exoskeleton prototype.

The tests in this paper were carried out by six volunteers (age: 22±1.8 years; weight: 70±4.5 Kg; height: 176±4.5 cm) [22,23], in which the testing procedure complied with the Declaration of Helsinki. To ensure the centralized completion of the test, two sets of measurement equipment were used, but to avoid measurement errors, a subject could only use one set of the respiratory metabolizer (Figure 14a) and exoskeleton (Figure 14b) to complete his test. To avoid the psychological fluctuations caused by their inadaptation with the exoskeleton, the subjects were required to conduct a half-hour adaptation training after adjusting the exoskeleton to their best subjective experience according to their physiological state [12,23,24,30,31].

**Figure 15:**
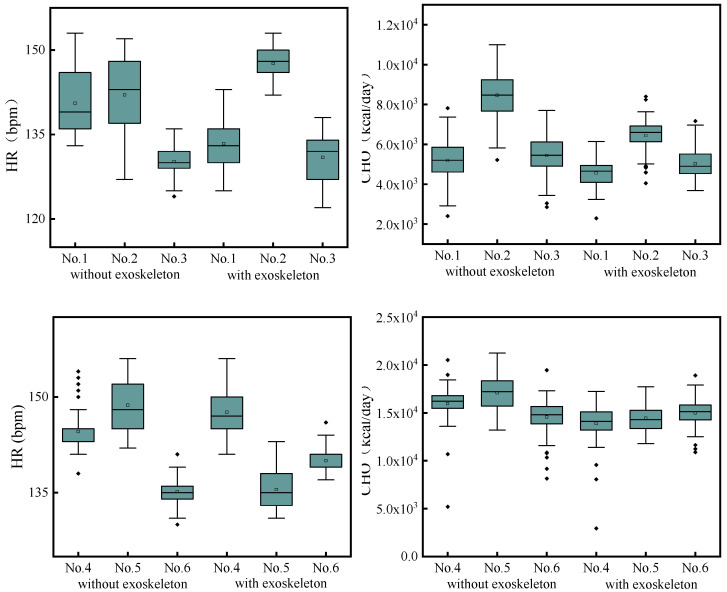
Measurement data under stationary motion.

Six volunteers were divided into two test groups. Each subject needed to conduct a complete test with and without the exoskeleton, and a rest time of more than 30 min should be guaranteed between the two tests of the same subject. The subjects in the same test group drew lots to determine the test sequence and were staggered to complete the test—after the completion of the test of the last subject, the subject who completed the rest progress or did not participate in the test started the test immediately. The measurement results are shown in Figure 15.

As shown in Figure 15, during the test, except for Subject No. 5, the heart rate of most subjects could be kept below 150 bpm, which shows that the tests in this paper ensure the validity of the indirect metabolic measurement. In addition, the subjects in the first group had lower heart rates in the tests with the exoskeleton than without the exoskeleton, while most subjects in the second group had higher heart rates in the tests with the exoskeleton than without the exoskeleton, which may be related to the psychological factors during the test environment, such as the nervousness, indicating that the adaptive training of the subjects in the second group did not achieve the desired effect or their interactions with the tester were not smooth and relaxed in the test [30,31].

Through the further analysis of the energy consumption data, the exoskeletons were shown to provide the walking assistance for more than 80% of the subjects. Especially, the exoskeletons provided greater walking assistance for Subject No. 2, provided a relatively average effect on Subjects No. 1, 4, and 5, but the effect on Subject No. 6 was not obvious, and it even provided the negative walking assistance. In the setting of the constant pace, the negative effect of the exoskeleton may be related to the psychological factors of the subjects or the increase of other consumptions including brain energy consumption, because the heart rate of Subject No. 6 increased significantly after wearing the exoskeleton. In addition, on the whole, the subjects had more concentrated distribution of energy consumption samples after wearing the exoskeleton, indicating that under the action of the exoskeleton, the human body has more uniform energy consumption, which is related to the homogenization of the joint torque after wearing the exoskeleton.

**Figure 16:**
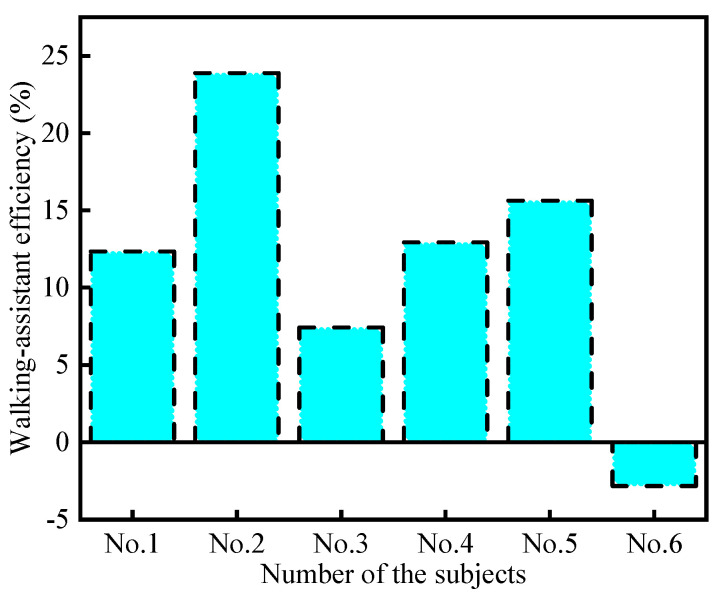
Walking-assistance efficiencies of the exoskeleton.

This paper further calculated the walking-assistance efficiencies provided by the exoskeleton to each subject, and the results are shown in Figure 16. The average of all positive walking-assistance efficiencies was calculated to be 14.45%, which is slightly higher than the previous exoskeleton tests [22,24,33].

## 7. Conclusions

Passive exoskeletons have advantages such as convenience, stability, and low cost, but their walking-assistance efficiencies were generally lower than powered exoskeletons [25]. In the existing exoskeleton design [4,23,24], the walking-assistance degree of the exoskeleton was ignored, which easily caused the exoskeleton to provide excessive torque to the human body, forcing the walking-assistance effect of the exoskeleton to become resistant to the human body, thereby reducing the walking-assistance efficiency of the exoskeleton. Therefore, based on the idea that selecting an optimal spring stiffness can produce an optimal metabolic benefit [3,23,25], this paper proposed a biomechanical-based cooperativity model to fill the technical gap in the exoskeleton design regarding the torque transmission law between humans and exoskeletons, aiming to improve the efficiency of the energy redistribution of the exoskeleton to the human body [26,27].

The cooperativity model was composed of the motion cooperativity and the torque cooperativity. In the torque cooperativity, the offset principle was firstly defined to clarify the overall trend of the working torque of the exoskeleton on the human body. Then, based on the walking-assistance characteristics of the hip and knee energy storage mechanisms, the cooperativity between the working torque and the joint torque was finally realized by proposing their average dispersion algorithms to optimize the parameters of the passive energy storage components and the parameters of the key exoskeleton structures. In addition, the concepts of three walking-assistance efficiencies (target walking-assistance efficiency, limit walking-assistance efficiency, and actual walking-assistance efficiency) and their relationship with each other were described, which indicated that the improvement of the walking-assistance efficiency brought by the increase of the scaling ratio of the joint torque was gradually eroded by the increase of the average dispersion degree between the joint torque and the working torque of the exoskeleton.

Due to the lack of objective comparative conditions among all existing exoskeletons, the effect of the cooperativity model on the exoskeleton was indicated by comparing the walking-assistance efficiencies of the exoskeletons with the same structure but with different elastic parameters of the energy storage components. The dynamic simulations showed that there exists and only one set of elastic parameters that can be set to obtain the maximum value of the walking-assistance efficiency, which is in line with the judgment of references [3,25] and the maximum values were 17.95% and 18.51% respectively for the hip joint and knee joint, which are generally higher than the previous lower-limb-assistant exoskeleton [3,4,23,24,30,31]. In addition, to actual test the walking-assistance efficiency of the exoskeleton with the optimal parameter combinations, the respiratory metabolisms with and without the exoskeleton were compared to calculate the efficiency, in which there is a regularity between the changes of the metabolic gases [22,24,33], and the exoskeleton provided the average walking-assistance efficiency of 14.45% for more than 80% of the subjects, which slightly higher than the previous-exoskeleton tests [22,24,33].

## 8. Limitations of the Study

In this paper, under the theoretical conditions, the good human–exoskeleton interaction, the cooperativity model was established to solve the optimal auxiliary conditions of the exoskeleton based on the model. Based on similar structures, the exoskeleton using the cooperativity model proved to have higher walking-assistance efficiency. However, although the exoskeletons are designed based on the cooperativity model, the measured walking-assistance efficiencies in Section 6 were lower than the simulated walking-assistance efficiencies in Section 5, which indicates the walking-assistance efficiency of the exoskeleton will decrease under the actual human–exoskeleton interaction conditions. The reasons are under realistic conditions the influence on the exoskeleton of the human–exoskeleton interaction form, the comfort factor and the friction between the exoskeleton and human, and the differences of the human joint torques, the sensitive condition analysis of the cooperativity model in the practical applications, which will be the focus of subsequent research.

In addition, to save processing cost, this paper defined a cubic equation to complete the design of the main cam. In the calculation process, it could be found that with the increase of the scaling ratio for the joint torque, the gap between the exoskeleton working torque and the joint torque changed significantly, which indicates that the cubic equation has poor tolerance to the joint torque in essence. To further play the role of the cooperativity model, we still need to define a more accurate cam curve.

## Data Availability

The datasets generated during and analyzed during the current study are available from the corresponding author on reasonable request.
